# Letter to the Editor: Integrative in silico analysis of per- and polyfluorinated alkyl substances (PFAS)-associated molecular alterations in human cancers: a multi-cancer framework for predicting toxicogenomic disruption

**DOI:** 10.1097/JS9.0000000000002858

**Published:** 2025-06-24

**Authors:** Deqian Xie, Zunwen Zheng, Chang Liu, Guangzhen Wu

**Affiliations:** a Department of Urology, the First Affiliated Hospital of Dalian Medical University, Dalian, China; b Department of thoracic surgery, Shenyang Tenth People’s Hospital/Shenyang Chest Hospital, Shenyang, China

*Dear Editor,*

We recently read the paper by Hong *et al* published in the *International Journal of Surgery* titled “Integrative in silico analysis of per- and polyfluorinated alkyl substances (PFAS)-associated molecular alterations in human cancers: a multi-cancer framework for predicting toxicogenomic disruption.” This study adopts an outstanding interdisciplinary perspective, creatively combining in vitro toxicogenomics with molecular docking simulations to systematically reveal the disruption of specific genes and signaling pathways following exposure to perfluorooctanoic acid (PFOA) and perfluorooctane sulfonic acid (PFOS) in six human cancer cell models. This provides important clues for understanding the causal relationship between environmental pollutants and tumorigenesis. The study balances both the breadth and depth of the data, by integrating methodologies such as protein-protein interaction networks, pathway enrichment, binding energy assessment, and toxicogenomic prediction, to construct a multi-level, multi-dimensional toxicity prediction framework. This framework reveals common molecular markers and specific responses of PFAS across different tissue contexts, enriching our understanding of PFAS toxicity mechanisms and laying a solid foundation for subsequent validation and clinical epidemiological studies^[1]^. The TITAN Guideline checklist was submitted according to the requirements of the literature “Transparency In the reporting of Artificial Intelligence-The TITAN guideline^[2]^.

Although the current study is comprehensive and outstanding, there are still some areas worthy of further exploration. The authors employed the Combat algorithm to correct batch effects across different TCGA cohorts and utilized the edge R tool for differential expression analysis, which represents standard statistical methodology. However, if future studies could visually demonstrate changes in sample clustering before and after correction, this would further assist readers in assessing the effectiveness of batch correction and mitigating potential biases^[3]^.

Increasing evidence suggests that exposure to PFAS affects epigenetic modification pathways. PFAS is closely associated with DNA methylation in human liver cells and miRNA dysregulation in breast cancer models^[4,5]^. Looking ahead, incorporating epigenomic and miRNA expression data into future analyses may reveal potential effects of PFAS on DNA methylation and non-coding RNA regulation. Combining spatial transcriptomics technology to observe the localization and expression heterogeneity of key genes in the tumor microenvironment may provide a more comprehensive directional guidance for elucidating PFAS-related carcinogenic effects. Additionally, if this toxicity prediction framework can be combined with PFAS exposure levels in real-world population samples and validated through cross-cohort epidemiological studies, it could further enhance the clinical translational value of this model in environmental risk assessment.

In summary, Hong *et al*’s study sets a new benchmark for PFAS toxicogenomics research with its innovative integrative approach, rigorous analytical workflow, and deep utilization of multi-omics data. We thank the *International Journal of Surgery* for providing a platform for the widespread dissemination of this research and sincerely look forward to the research team continuing to deepen their work in this field, driving related cancer research to new heights.

## Data Availability

The data that support the findings of this study are all included in this article.
